# 25, 50 and 75 Years Ago

**DOI:** 10.1111/ans.70191

**Published:** 2025-05-24

**Authors:** Julian A. Smith

**Affiliations:** ^1^ Department of Surgery Monash University Melbourne Victoria Australia

## Twenty‐Five Years Ago

**Margovsky A. Unplanned Admissions in Day‐Case Surgery as a Clinical Indicator for Quality Assurance. ANZ J Surg. 2000;70:216–220**.

Day surgery is a modern, effective, and economical way to treat patients while maintaining the same level of quality of patient care. Quality improvement in day surgery units, however, continues to be an issue due to high rates of unplanned admissions. The aim of the present retrospective study was to investigate reasons for and methods of preventing unplanned postoperative admissions in a day surgical unit over a 12‐month period in respect to different surgical specialties. The study was based on an audit from the Endoscopy and Day Surgery Unit (EDSU) at Launceston General Hospital, which provides health care to a population of more than 120 000. For the accounted period, 920 outpatients had elective day surgical procedures. Overall, the unplanned admission rate was 4.7%, and surgical, anaesthetic, and social reasons accounted for 58.2%, 37.2%, and 4.6% of the unplanned admissions, respectively. The highest rate of unplanned admissions was for plastic and reconstructive surgery (12.8%) and orthopaedic surgery (7.5%) despite the relatively small number of patients who underwent such procedures in the day surgery unit. The results also showed a correlation between age group, pre‐operative medical status of the patients found suitable for the day surgical procedure, and unplanned admissions. Strategies to reduce the unplanned admission rate, which include patient selection and pre‐operative assessment, patient waiting time and education, pre‐operative anaesthesia, follow‐up with nursing care, and postoperative analgesia, are discussed.

**Mills SJC, Holland DJ, Hardy AE. Operative Field Contamination by the Sweating Surgeon. ANZ J Surg. 2000;70:837–839**.

There are a number of factors relating to the host, bacteria, and wound that are important in the development of wound infection. The effect of the surgeon sweating has not been previously reported. Ten surgeons performed a mock total hip joint operation under sterile conditions while not sweating and then repeated the operation while sweating. Settle plates were used to quantify the bacterial counts in the operative field in both phases. For each subject, a mean of 3.3 colony forming units (c.f.u.) was present in the non‐sweating phase and 6.9 c.f.u. were present in the sweating phase (*p* < 0.05), organisms grown were normal skin flora. The sweating surgeon may be more likely to contaminate the surgical field than the non‐sweating surgeon. It is important for orthopaedic surgeons, especially those performing joint replacement surgery, to be aware of this and to take measures to minimize sweating in the operating theatre.

## Fifty Years Ago

**Britten‐Jones R. A Major Advance in the Management of Pneumatosis Coli. ANZ J Surg. 1975;45:367–369**.

A new treatment for gas cysts of the large bowel is described which involves the continuous inhalation of a high concentration of oxygen over a 5‐day period. Two patients with incapacitating symptoms due to diffuse pneumatosis coli were treated by this method. Oxygen therapy resulted in remission of symptoms and disappearance of cysts in both cases. The physiological basis of this simple, effective therapy is discussed, together with the precautions necessary in its use. The two patients reported have remained free of symptoms for 15 and 4 months respectively, but even if recurrence occurs, it would seem that further courses of oxygen treatment can be given with prolonged symptomatic relief. If used with caution, this is a safe, simple, and effective method of treatment of pneumatosis coli causing severe symptoms in patients who previously could be offered only symptomatic treatment or major excisional surgery.

**Little JM, Shiel AGR, Loewenthal J, May J, Goodman AH. Mean Flow Measurements in Aortofemoral Arterial Reconstructions. ANZ J Surg. 1975;45:17–21**.

Eighty‐seven limbs in 48 patients have been studied with an electromagnetic flow meter at the time of arterial reconstruction designed to restore blood flow from the aorta to the femoral arterial system. The mean overall flow was 339 mL per minute. Blood flow in aneurysmal disease was significantly higher than that recorded in obstructive disease. Blood flow in arteries running into a fully patent femoral system was significantly higher than in those that ran only into the profunda. Age, sex, type of surgery, and size of graft had no influence on the flow achieved. Reconstructions for rest pain or advanced trophic change produced flows of the same magnitude as reconstructions for intermittent claudication. The patient's weight correlated significantly with the intraoperative flow. The mean flow in the limbs of 28 patients in whom an accurate preoperative weight was known was 2.56 mL/100 g/min, taking the hind limb and hemi‐pelvis as 25% of the body weight. This is an acceptably ‘normal’ value. A flow of less than 100 mL per minute in an aortoiliac reconstruction was found to be not likely to result in long‐term patency.

## Seventy‐Five Years Ago

**King ESJ. The Genesis of Varicose Veins. ANZ J Surg. 1950;20:126–133**.

In review, at the early stage of varicosis there is in an area, a dilatation, sometimes of sudden onset, without valvular incompetence, with centripetal pulsation and without involvement of the proximal part of the veins. At this stage, the veins are capable of contraction, that is, the muscle is not degenerate nor intrinsically weak; yet dilatation does occur. These phenomena are explained by the action of some chemical factor, possibly a hormone or hormones of the oestrogen group, which have been shown to produce relaxation of smooth muscle in other tubes.

Once the condition has developed, then, just as with other structures such as bones, mechanical factors come into play and produce the various secondary changes which are clearly the effect of hydrostatic stresses. These give rise to the structures which are ‘ill‐faced, worse bodied, shapeless everywhere’. The distinction between the two stages is of paramount importance in distinguishing between primary aetiological factors and those responsible for the more obvious and, in some respects, the more important secondary changes.

